# SARS-CoV-2 antibody prevalence in health care workers: Preliminary report of a single center study

**DOI:** 10.1371/journal.pone.0240006

**Published:** 2020-11-12

**Authors:** Michael Brant-Zawadzki, Deborah Fridman, Philip A. Robinson, Matthew Zahn, Clayton Chau, Randy German, Marcus Breit, Jason R. Bock, Junko Hara

**Affiliations:** 1 Hoag Center for Research and Education, Hoag Memorial Hospital Presbyterian, Newport Beach, California, United States of America; 2 Infection Prevention, Hoag Memorial Hospital Presbyterian, Newport Beach, California, United States of America; 3 Orange County Health Care Agency, Santa Ana, California, United States of America; 4 Laboratory Administrative Services, Hoag Memorial Hospital Presbyterian, Newport Beach, California, United States of America; 5 Hoag Family Cancer Institute, Hoag Memorial Hospital Presbyterian, Newport Beach, California, United States of America; 6 Medical Care Corporation, Newport Beach, California, United States of America; University of Auckland, NEW ZEALAND

## Abstract

Serological surveys have been conducted to establish prevalence for COVID-19 antibodies in various cohorts and communities, reporting a wide range of outcomes. The prevalence of such antibodies among healthcare workers, presumed at higher risk for infection, has been increasingly investigated, more studies are needed to better understand the risks and infection transmission in different healthcare settings. The present study reports on initial sero-surveillance conducted on healthcare workers at a regional hospital system in Orange County, California, during May and June, 2020. Study subjects were recruited from the entire hospital employee workforce and the independent medical staff. Data were collected for job duties and locations, COVID-19 symptoms, a PCR test history, travel record since January 2020, and existence of household contacts with COVID-19. A blood sample was collected from each subject for serum analysis for IgG antibodies to SARS-CoV-2. Of 2,992 tested individuals, a total 2,924 with complete data were included in the analysis. Observed prevalence of 1.06% (31 antibody positive cases), adjusted prevalence of 1.13% for test sensitivity and specificity were identified. Significant group differences between positive vs. negative were observed for age (z = 2.65, *p* = .008), race (*p* = .037), presence of fever (*p* < .001), and loss of smell (*p* < .001), but not for occupations (*p* = .710). Possible explanation for this low prevalence includes a relatively low local geographic community prevalence (~4.4%) at the time of testing, the hospital’s timely procurement of personal protective equipment, rigorous employee education, patient triage, and treatment protocol development and implementation. In addition, cross-reactive adaptive T cell mediated immunity, as recently described, may possibly play a greater role in healthcare workers than in the general population.

## Introduction

SARS-CoV-2 has driven a pandemic crisis. Its hallmark is very high infectivity, pre-symptomatic transmission and asymptomatic prevalence which continue to fuel dramatic cumulative numbers of infections, hospitalizations, and deaths. To better understand the extent of undetected transmission, serological surveys in sampled cohorts identified antibodies from prior infection ranging from 57% prevalence in Bergamo—Italy’s epicenter [1], 20% in New York City [2], 5.2% in Kenya [3], down to 4.7% in Los Angeles County [4] and 2.8% in Santa Clara County [5], California.

While prevalence of antibodies among healthcare workers, presumed at high risk for infection, has also been increasingly studied, the prevalence, sample size, and sampling methodology greatly varies. Garcia-Basteiro, et al reports the cumulative prevalence (IgG, IgA, or current positive rRT-PCR) of SARS-CoV-2 infection of 11.2% among 578 subjects in a large hospital in Spain [6]. The study of 28,792 healthcare works from Denmark identified 2.81% prevalence for IgM and 2.67% for IgG, and found higher prevalence in front-line workers specifically working with COVID-19 patients, compared to other front-line workers [7]. It also found that subjects younger than 30 years had the highest seroprevalence compared to those who are 30 years or older. Another study found 13.7% IgG prevalence among 40,329 healthcare workers in the greater New York city area [8] similar to the community prevalence in New York State (14.0%) [9], and sero-positivity was strongly associated with self-reported suspicion of prior COVID-19 exposure and prior positive PCR testing.

Further determining such prevalence among healthcare workers in varied geographic areas and examining duration of antibody presence may help stratify the workforce for risk, establish better health place policies and procedures, and potentially better mitigate transmission across different healthcare settings.

This article reports on initial sero-surveillance conducted among 2,992 healthcare workers at Hoag Memorial Hospital Presbyterian, a regional hospital system in Orange County, California, United States, during May and June, 2020.

## Methods

### Recruitment and enrollment

The Institutional Review Board approval was obtained for this study from Providence St. Joseph Health (IRB # 2020000337). Study subjects were recruited by email notifications to the entire employee workforce (6000+ individuals) and the independent medical staff (1600+ physicians), and were enrolled during May and June, 2020. Their work locations included 2 main hospital campuses, 9 health centers, 13 urge care locations, and other clinical and administrative facilities all within approximately 20 miles radius. Informed consent was obtained in person originally (n = 2,934), then electronically after June 18, 2020 (n = 58). All consenting subjects were asked to answer a questionnaire (S1 Table), and a blood sample was collected.

### Questionnaire

The questionnaire was developed by two of the physician authors, one of whom is an infectious disease specialist, within the guidelines of the human resources department of Hoag Hospital and the rules and regulations of the Hoag medical staff department. The goal of the questionnaire development was ease of response while allowing collection of basic information including demographics, job duties and locations, and potential outside exposures. In addition, the COVID-19 symptoms were selected based on the list of symptoms by CDC and other COVID-19-related publications at the time of questionnaire development.

Using the reported job duties and locations, each subject was classified into a) high (e.g., MD, RN, PA, emergency care tech, ICU tech), b) medium (e.g., therapist, phlebotomist, medical tech), or c) low (e.g., administration, coding, billing, lab tech/scientist, IT) risk groups to approximate levels of direct exposure to COVID-19 patients.

### IgG antibodies to SARS-CoV-2 analysis

A 5 ml peripheral draw venous blood sample was collected, at the time of in-person consent and within 7 days (*M* = 1.67, *SD* = 1.36) of electronic consent, from each subject into a gold top serum separator vacutainer tube (BD Medical). Samples were centrifuged within 2 hours of collection at 4500 RPM for 5 minutes (RCF 3060). Aliquots were analyzed with calibrated lots of Anti-SARS-CoV-2 IgG Reagent Pack on the VITROS® XT 7600 according to manufacturer’s instructions for use. SARS-CoV-2 spike protein coated on lots is the antigen used [10]. Positive and negative quality controls were run daily prior to sample analysis (Ortho Diagnostics Anti-SARS-CoV-2 IgG Control). At the time of writing, this IgG test was approved only for use under the Food and Drug Administration’s Emergency Use Authorization.

Manufacture sensitivity and specificity claims for the Ortho Clinical Diagnostics VITROS Anti-SARS-CoV-2 IgG assay is 100% (407/407) negative agreement (95% CI: 99.1–100.0%) in 407 presumed SARS-CoV-2 antibody negative subjects and 87.5% (42/48) positive agreement (95% CI: 74.8–95.3%) in 48 PCR positive subjects with days from positive PCR ranging from 1 day to 22 days and days from onset of symptoms ranging from 12 to 32 days. In-house validation studies were conducted with 35 samples from subjects with a known positive SARS-CoV-2 PCR test a mean of 43 days out from positive PCR test date (range 38–48 days), and 50 samples from subjects with a known negative SARS-CoV-2 PCR test. Twenty-nine of 31 PCR samples were positive for SARS-CoV-2 IgG antibody. All 50 of the PCR negative samples were SARS-CoV-2 IgG antibody negative. Thus, sensitivity of 93.6% (95%CI: 78.6–99.2%) and specificity of 100% (95% CI: 92.9–100.0%) were calculated for the Ortho Diagnostics VITROS Anti-SARS-CoV-2 IgG assay in run our laboratory on the Ortho Clinical Diagnostics VITROS® XT 7600 automated instrument platform, and adopted in this study.

### Data analysis

Demographic, occupational, and symptom factors were assessed for group differences between negative vs. positive for the presence of IgG antibodies. A Mann-Whitney U test was used for assessing group difference in age, and a series of Fisher’s exact tests were used for the remaining categorical factors; for group differences in race (a 7×2 table), the Mehta-Patel algorithm [11] was applied. A value of *p* < .05 was used for statistical significance. For all analyses, the Stata statistical software package, edition 15 [12], was used.

## Results

Of an initial 2,992 samples recorded, subjects were excluded from analyses due to missing age (n = 3), gender (n = 14), race, (n = 31), occupation (n = 8), and symptoms (n = 12), resulting in a complete pool of 2,924 (Table 1).

**Table 1:** Sample characteristics and group differences.

	Antibody Negative	Antibody Positive	Total	
	n = 2893 (98.9%)	n = 31 (1.06%)	N = 2924 (100%)	*p*
Age in yrs., *M* (*SD*)	42.67 (12.10)	37.58 (12.30)	42.62 (12.12)	.008
Female, count (%)	2097 (72%)	23 (74%)	2120 (72%)	.508
Race, count (%)				.037
American Indian or Alaska Native	19 (1%)	0	19 (1%)	
Asian	653 (23%)	10 (32%)	663 (23%)	
Black	47 (2%)	0	47 (2%)	
Hispanic or Latino	485 (17%)	11 (35%)	496 (17%)	
Native Hawaiian or Pacific Islander	50 (2%)	1 (3%)	51 (2%)	
White	1455 (50%)	9 (29%)	1464 (50%)	
Other	184 (6%)	0	184 (6%)	
Occupational risk level, count (%)				.710
Low	769 (27%)	6 (19%)	775 (27%)	
Medium	535 (18%)	6 (19%)	541 (19%)	
High	1589 (55%)	19 (61%)	1608 (55%)	
Fever, count (%)	331 (11%)	12 (39%)	343 (12%)	< .001
Cough, count (%)	473 (16%)	7 (23%)	480 (16%)	.332
Sore Throat, count (%)	550 (19%)	7 (23%)	557 (19%)	.645
Runny Nose, count (%)	403 (14%)	7 (23%)	410 (14%)	.188
Loss of Smell, count (%)	55 (2%)	13 (42%)	68 (2%)	< .001

Note. Group difference testing was performed with Mann-Whitney U tests for age and with Fisher's exact tests for categorical measures.

Antibody testing identified 31 positive cases (2,893 negative), resulting in an observed prevalence of 1.06% (exact binomial 95% CI = 0.72% - 1.50%). Accounting for test sensitivity of 93.6% and specificity of 100%, an adjusted prevalence of 1.13% (95% CI = 0.78% - 1.58%) was calculated, indicating 33 positive cases (negative = 2,891) after adjustment.

Nonparametric tests for group differences were performed for demographics and five symptoms of COVID-19. Significant differences between observed negative and positive cases were found for age (z = 2.64, *p* = .008), race (*p* = .037), presence of fever (*p* < .001), and loss of smell (*p* < .001), but not for occupation (*p* = .710). Interestingly, of those with previously PCR confirmed diagnosis of COVID-19 (n = 11), 6 were antibody positive with 5 non-reactive. None of the non-reactive 5 had a history of hospitalization or severe illness.

## Discussion

Our study found a significantly lower prevalence (1.06% observed prevalence) of SARS-CoV-2 antibody carriers among our healthcare workers compared to prior reports ranging from 2.6% to 13.7%. During this same period, prevalence of antibodies tested by physician order at our hospital laboratory was 3.87%.

One possible explanation for the low seroconversion rate in our work force is a relatively low overall regional estimated prevalence of infections (~4.4%), as further evidenced by an average 104 patients per day in ICU and 330 cumulative deaths in Orange County (total population of 3.18 million) at the time of our study [13]. This hypothesis is supported by the considerably higher prevalence in healthcare workers and even higher community prevalence in New York [9], indicating that geographic consideration needs to be given when evaluating the infection and transmission risks among healthcare workers.

Despite our relatively low community prevalence in the early stage of the pandemic, our institution had implemented stringent workforce education on personal hygiene, social distancing and appropriate PPE usage since January 2020 when we saw the first California and third US case, with hospital-wide protocols in patient triage and symptom surveillance. Such strategies may have heightened our healthcare workers’ awareness, urgency, and compliance with our policies, both at and outside work place, possibly contributing to the lower prevalence we have found in this study.

In addition, recent research indicates presence of innate and cross-reactive adaptive T cell mediated immunity, which may lower susceptibility to COVID-19 infection in some individuals. One might speculate that greater frequency of exposure to such agents occurs in healthcare workers vs. the general population. Several studies have reported that such innate T cell immunity exists [14] with documented cross-reactivity to related corona virus species [15–17]. One can speculate that workplace exposure is more frequent for health care workers to such various coronavirus pathogens. A combination of all of the above factors may explain our findings.

In our cohort, there were 11 cases with previously PCR confirmed diagnosis of COVID-19 with 5 non-reactive cases, which cannot be fully explained by antibody test sensitivity and specificity. Recent studies found a rapid decay of IgG antibody in patients with mild symptoms, with the possible span of 2–3 months [18, 19]. Of those 5 non-relative cases, 4 reported no or mild COVID-19 symptoms in the questionnaire, their confirmed COVID-19 results being up to 2 months prior to antibody tests. While this provides additional support for the recent findings [17, 18], further research in the larger cohort is needed, and whether such decrease in antibodies lowers immunity should be examined, given the extremely rare cases of re-infection being reported [20].

We will retest this same cohort at 8 weeks and 6 months, to better understand the dynamics of SARS-CoV-2 antibody prevalence and duration in healthcare workers.

## Supporting information

S1 Table Study questionnaire. (DOCX)
