# Supplementary material for: SARS-CoV-2 antibody prevalence in health care workers: Preliminary report of a single center study
Source: PLoS One. 2020 Nov 12;15(11):e0240006. doi: 10.1371/journal.pone.0240006 (PMC7660494; doi:10.1371/journal.pone.0240006)
Supplement: S1 Table — (DOCX) [file pone.0240006.s001.docx]

**SARS-CoV-2 Antibody Test Collection Form**

First Name_________________________________ Last Name_________________________________

DOB (mm/dd/yyyy) ___________________ SS#: ___________________________________

Hoag E-mail Address ________________________________________________________

(or alternative e-mail address for notification of results)

Check if you work for : Police or Fire

Job Title _______________________________________________________

Job Location __________________________________________________

Race

American Indian or Alaska Native

Asian

Black

Hispanic or Latino

Native Hawaiian or Pacific Islander

White

Other

Covid Questions:

- Tested positive for COVID within the last 14 days?
  - Yes No
  - If “Yes”, date of positive test ________________
- Any of the following symptoms since January?
  - Fever
  - Sore throat
  - Cough
  - Runny Nose

Loss of Smell

- Travelled outside of California since January to conventions, resorts, large sporting events, family gatherings, other meetings?
  - Yes No
- Come in close contact with anyone outside of the workplace who developed a confirmed COVID illness that you are aware of
  - Yes No

I have received a copy of the Antibody Testing Information Sheet.

Signature: ______________________________________________ Date: ________________________
